# Sleep in Normal Aging, Homeostatic and Circadian Regulation and Vulnerability to Sleep Deprivation

**DOI:** 10.3390/brainsci11081003

**Published:** 2021-07-29

**Authors:** Jacques Taillard, Claude Gronfier, Stéphanie Bioulac, Pierre Philip, Patricia Sagaspe

**Affiliations:** 1 Sommeil, Addiction et Neuropsychiatrie, Université de Bordeaux, SANPSY, USR 3413, F-33000 Bordeaux, France; stephanie.bioulac@chu-bordeaux.fr (S.B.); pierre.philip@u-bordeaux.fr (P.P.); patricia.sagaspe@chu-bordeaux.fr (P.S.); 2 CNRS, SANPSY, USR 3413, F-33000 Bordeaux, France; 3 Lyon Neuroscience Research Center (CRNL), Integrative Physiology of the Brain Arousal Systems (Waking) Team, Inserm UMRS 1028, CNRS UMR 5292, Université Claude Bernard Lyon 1, Université de Lyon, F-69000 Lyon, France; claude.gronfier@inserm.fr; 4 Pôle Neurosciences Cliniques, CHU de Bordeaux, F-33076 Bordeaux, France

**Keywords:** normal aging, sleep, circadian rhythms, sleep homeostasis, sensitivity to light, cognitive performance, sleepiness, differential vulnerability

## Abstract

In the context of geriatric research, a growing body of evidence links normal age-related changes in sleep with many adverse health outcomes, especially a decline in cognition in older adults. The most important sleep alterations that continue to worsen after 60 years involve sleep timing, (especially early wake time, phase advance), sleep maintenance (continuity of sleep interrupted by numerous awakenings) and reduced amount of sigma activity (during non-rapid eye movement (NREM) sleep) associated with modifications of sleep spindle characteristics (density, amplitude, frequency) and spindle–Slow Wave coupling. After 60 years, there is a very clear gender-dependent deterioration in sleep. Even if there are degradations of sleep after 60 years, daytime wake level and especially daytime sleepiness is not modified with age. On the other hand, under sleep deprivation condition, older adults show smaller cognitive impairments than younger adults, suggesting an age-related lower vulnerability to extended wakefulness. These sleep and cognitive age-related modifications would be due to a reduced homeostatic drive and consequently a reduced sleep need, an attenuation of circadian drive (reduction of sleep forbidden zone in late afternoon and wake forbidden zone in early morning), a modification of the interaction of the circadian and homeostatic processes and/or an alteration of subcortical structures involved in generation of circadian and homeostatic drive, or connections to the cerebral cortex with age. The modifications and interactions of these two processes with age are still uncertain, and still require further investigation. The understanding of the respective contribution of circadian and homeostatic processes in the regulation of neurobehavioral function with aging present a challenge for improving health, management of cognitive decline and potential early chronobiological or sleep-wake interventions.

## 1. Introduction

Determining the normal age-related changes that occur in sleep, especially in the elderly (over 60 years old), and their cause is of interest in public health since, about 50% of older adults complain about having difficulty in initiating or maintaining sleep [1], and that worse sleep is associated with worse health, quality of life and cognitive decline. Patel et al. [2] confirmed in a review that 30–48% of elderly subjects complain of insomnia symptoms, but only 10–20% suffer from an insomnia disorder (sleep difficulties present for ≥3 nights per week and lasting for >3 months, depending on the diagnostic criteria). In addition, it is now often accepted that normal sleep in the elderly is objectively characterized by difficulties in sleep timing, initiation and maintenance associated with a modification of the macro and microarchitecture of sleep [3]. However, a meta-analysis (65 studies, 2391 adults, sleep parameters measured by objective measures: polysomnography) in 2004 demonstrated that most age-dependent changes in sleep architecture occur before the age of 60 years [4]. The most important sleep alterations that continue to worsen after 60 years involve sleep timing (especially early wake time), sleep maintenance (sleep interrupted by numerous awakenings) and reduced amount of slow wave activity (during deeper sleep). These sleep modifications could be associated with changes in homeostatic (sleep pressure) and circadian (biological clocks) factors, the key physiological drivers of sleep regulation.

This difference between the results of meta-analyses, clinical studies and sleep complaints in elderly subjects may be explained in several ways. First, determining sleep patterns in normal aging is a challenging task because sleep pathologies (nocturnal respiratory disorders or abnormal movement during sleep) or comorbidities (medical and psychiatric) and medications/substances that have an impact on sleep structure (cardiovascular, metabolic, psychiatric or neurological diseases and pain) are highly prevalent in elderly people. Secondly, there are very few meta-analyses, and to our knowledge, few longitudinal studies that provide a full understanding of age-dependent changes in sleep. Finally, there are sex differences in age-related changes in sleep/wake cycle [5], a factor not considered in most studies. These methodological challenges make it difficult to determine the exact normal age-related changes in sleep/wake cycle. However, big data analysis using wearable devices in the general population can provide better information on the evolution of sleep timing, duration and maintenance with age, especially in elderly subjects [6].

Even if sleep complaints in the elderly are often associated with comorbidities or medication, changes in the sleep-wake cycle are also observed in healthy subjects. This review focuses on changes in the regulatory processes of the sleep/wake cycle related to normal aging rather related with pathological process. First, we review modifications in sleep timing, the initiation and maintenance of sleep in normal aging after 60 years and the modifications that occur in its macro- and micro-architecture. Then we characterize the basics of sleep wake regulation, the interactions between homeostatic and circadian processes and the modifications associated with normal aging. Finally, we focus on daytime sleepiness, cognition and vulnerability to sleep deprivation in older subjects.

## 2. Age-Related Changes in Sleep Organization and Structure

### 2.1. Sleep Timing

Sleep timing (assessed by bedtime, wake time or midsleep) shifts towards an earlier time with aging even after 60 years of age. Older adults go to bed earlier in the evening, and wake up approximately 1–2 h earlier in the morning than younger adults [7]. This phase advance has been confirmed in large cohorts of subjects [8,9,10]. These studies also demonstrated a sex difference in sleep timing: after the age of 60, women show on average a later chronotype than men, but this sex difference disappears again at around 75 years. In a longitudinal study, Didikoglu et al. [11] confirmed this phase advance in more than 6000 participants with a long follow-up period (up to 35.5 years). The average midsleep time was predicted to be 3:35 a.m. before age 60 and 3:21 a.m. after age 80. The sleep midpoint declined approximately 6 min for each decade, and shifted 30 min earlier from age 40 to 90+ years. In a study analyzing 1.14 million nights from wearable activity trackers in more than 69,000 adult non-shift workers aged between 19–67 years from 47 countries, the authors demonstrated that sleep onset is earlier with age, and that women fall asleep earlier than men. However, this difference no longer exists after 60 years, with people falling asleep earlier during weekdays than weekends, a tendency that is more pronounced with age in men [6]. After 60 years, women fall asleep later than men. With age, people tend to wake up earlier with a one-hour difference between weekends and weekdays across their lifespan. After 60 years, women wake up later than men during weekends. Interestingly, weekdays sleep offset varies by country; a weekday wake up timing in late adulthood was apparent in Germany and the United Kingdom, but was not in Japan.

### 2.2. Sleep Duration

Although the National Sleep Foundation (NSF) recommends 7 to 8 h of adequate sleep for older adults, this is one hour less than for adults aged 26 to 64 years [12]. A meta-analysis demonstrated that total sleep time (TST) declines with age until 60 years, and that it does not continue to decline significantly thereafter [4]. In a small group of healthy subjects (*n* = 36), Mander et al. [13] found that TST continued to decease after 60 years. In fact, the NSF recommendation is supported by evidence that sleep lasting 6 to 9 h in older adults leads to better cognition, health and quality of life. On the other hand, studies in a natural setting showed that objective and subjective sleep duration increases after age 60 years. In a large Scandinavian cohort (*n* = 21,000), subjective weekday sleep duration but not weekend sleep duration increased from the age of 60–64 years to reach a plateau from the age of 70–74 years [14]. This difference was confirmed by a big data analysis of data obtained with wearable devices [6]. From the age of 65 years, the difference between weekdays and weekends no longer existed. Sleep duration was greater in women than in men [6,14]. This gender difference in sleep duration was already observed by Ohayon et al. [4] in a meta-analysis: effect sizes calculated for sex indicated that women had a longer TST than similarly aged men. Based on the PSG of more than 1100 subjects without sleep disorders (aged 40–80 years), Luca et al. [15] found that sleep duration decreased with age (but no information was given about whether sleep duration decreased significantly after 60 years) in both men and women. Based on the PSG of more than 1000 subjects (aged 20–80 years), Moraes et al. [16] found that sleep duration decreased with age (but no information was given about whether sleep duration decreased significantly after 60 years), especially in women. By compiling PSG data from two cohorts, the Multi-Ethnic Study of Atherosclerosis (MESA, *n* = 1595, 54–93 years of age, 48% were male) and the Osteoporotic Fractures in Men Study (MrOS, *n* = 2224 males, 67–96 years of age), Djonlagic et al. [17] demonstrated that older men have a shorter TST than older women. 

### 2.3. Sleep Initiation and Maintenance

Sleep initiation is measured by sleep latency (lights out to the first epoch of any sleep, expressed in minutes) and to fall back to sleep after nocturnal awakening. Several studies were unable to demonstrate any clear age effect in sleep latency [4,13,15,18], even though it is commonly accepted that the initiation of sleep deteriorates with advancing age [3]. Women have longer sleep latency than men [4], regardless of their age; this means that women find more difficulty in falling asleep than men. In experimental conditions (bedrest and forced desynchrony), Klerman et al. [19,20] demonstrated that older subjects had no greater difficulty falling back to sleep after a spontaneous awaking, but the likelihood of awakening from sleep—specifically during NREM sleep—was much greater in older than in young adults [20]. 

On the other hand, there is a consensus that sleep maintenance continues to deteriorate significantly after the age of 60 and beyond the age of 90 [4,13,15,17,21]. Sleep efficiency (ratio of total sleep time to time in bed expressed in percent, expressing the percentage of time that a person sleeps from bedtime to morning wake time) decreases with age, even after 60 years. Ohayon et al. [4] demonstrated in their meta-analysis that effect size was larger in women than in men, indicating a greater association between declining sleep efficiency and aging among women. On the other hand, Luca et al. [15] did not confirm this association, and Moraes et al. [16] found the opposite (larger effect size in men). In accordance with the results of the Sleep Heart Health Study (SHHS, 2685 participants, aged 37 to 92 years), after adjusting for significant factors, especially comorbidities, women have a worse sleep efficiency and spend a longer period asleep than men [22]. Wake after sleep onset (WASO, awakening duration after sleep onset, expressed in minutes) increases throughout life until the age of 60 years [4]. A PSG study in more than 1000 subjects found similar results, but the authors observed an increase in WASO after 74 years. In the compilation of the data from the MESA and MrOs cohorts, Djonlagic et al. [17] demonstrated that WASO increases with age (even after 60 years). WASO consistently increased about 10 min per decade of age from 30 years [4]. Luca et al. [15] found that WASO increases with age (but no information was given whether WASO decreases significantly after 60 years) in both men and women. Jonasdottir [6] found that the percentage of people with non-zero median WASO increased with age, and that that it was greater in women up to the age of 40, after which there was no difference between the sexes. On the other hand, Moraes et al. [16] observed larger effect sizes for WASO in men, with an increase with age. 

Sleep continuity can be assessed by the number of arousals (brief arousals during sleep lasting 3 to 14 s). Arousals are abrupt shifts in EEG frequency, including alpha, theta and/or frequencies greater than 16hz that last 3 s, with at least 10 s of stable sleep preceding the change. During REM, a concurrent increase in submental EEM lasting at least 1 s is mandatory [23]. The number of arousals increases with age even in subjects without sleep disorders, but does not continue to increase after 60 years [15,16,22,24]. Concerning changes in sleep-stage transitions, there are more transitions to and from the wake stage with normal ageing. In particular, the number of transitions between NREM (Non rapid eye movement = N1, N2 an N3 sleep stage) and REM (rapid eye movement) sleep stages relative to the number of transitions to wake is approximately six times higher in young persons than in older ones, highlighting the difficulty in maintaining sleep in older persons [20]. With normal ageing, there is an increase in the number of transitions between light sleep stages N1 and N2 and between N1 and W [25]. 

### 2.4. Macro-Architecture/Sleep Stages

Concerning NREM sleep, the percentage of stage N1 and N2 increases and stage N3 (deep sleep, slow wave sleep) decreases with age, but does not change after 60 years [4,16,26]. Djonlagic [17] demonstrated that these sleep stage modifications continue to evolve even after 60 years. Stage N1 and N2 but not N3 differ between mid- and late life [21]. A gender difference in age-related changes in sleep stages was found, with men having a lower percentage of stage N3 [4,16,17] and a higher percentage of stage N2 [4]. On the other hand, the results of the SHHS cohort demonstrated that age-related changes in sleep architecture depend mostly on gender, with marked sleep differences in men but not in women. While a progressive reduction in the percentage of stage N3 and a progressive increase in stages N1 and N2 are seen with increasing age in men, no consistent variation in the percentage of stages N1, N2 or N3 with age was observed in women [22]. In any case, all these gender differences suggest a gender-dependent deterioration in NREM.

While the percentage of stage REM sleep decreases until 60 years [4,16,21,26], REM latency is not modified with age [4]. In a meta-analysis focusing only on REM percentage, Floyd et al. demonstrated that the percentage of REM decreases slowly until age 75 (linear decrease in the proportion of REM with a small rate of 0.6% per decade from age 19 until 75 years) and then increases slowly until age 85 [27]. Gender differences have been observed in many studies [4,16,17], also suggesting a gender-dependent deterioration in REM.

### 2.5. Micro-Architecture/Sleep Patterns

Micro-architecture/sleep patterns and classical parameters of sleep are calculated by a visual stage-of-sleep analysis of sleep EEGs in accordance with AASM scoring rules for polysomnography [23]. Microstructure determines the EEG frequency (slow wave activity or delta, theta, alpha, sigma activity) and/or the quality and quantity of phasic EEG activity during sleep as spindles or K-complexes. Generally, the principal way to study microarchitecture is to analyze EEG in the time (evolution over time and space) and frequency domain (describing the same time frequency and amplitude of the signal). Analyses based on the frequency domain are the most widely used, especially the Fast Fourier Transform (FFT). Time-frequency domain (in both time and frequency domain) methods like wavelet transform are sometimes used to characterize sleep microarchitecture. 

#### 2.5.1. Whole-Sleep EEG Power Spectral Density

Compared to younger adults, power reductions ranging between 3 and 5 dB have been observed in sleep EEG recordings in middle-aged adults, with a further reduction of 5 dB in elderly subjects above the age of 80 [17]. Interestingly, absolute NREM spectral power is higher in women than in age-matched men. This sex difference is less pronounced when spectral power is expressed as relative power [15], perhaps due only to a difference in skull thickness. 

#### 2.5.2. Slow Wave Activity (SWA 0.5–4.5 Hz) in NREM Sleep

The decrease in SWA is the major sleep modification that occurs with age [3,13,15,17,21,28,29,30,31,32]. This age-related decrease in SWA is observed over the prefrontal cortex area and in the first NREM sleep cycles. While Djik et al. [26] did not find any decrease in SWA after 60 years, Luca et al. [15], Mander [13] and Djonlagic [17] found that SWA decreased until the age of 96 years. On the other hand, slow power (<1 Hz) does not decrease with age [17]. Dubé et al. [33] found that older subjects (mean age = 60 y) showed lower slow wave (SW) density (number of slow waves per minute of NREM sleep), amplitude (difference in voltage between negative and positive peaks) and slope (between the positive and the negative peaks in microvolts per second), especially in frontal sites. Djonlagic et al. [17] confirmed these results and demonstrated that SW duration increases with age, meaning that SWs become slower with age. In accordance with macro-architecture analysis, older women had higher absolute SWA spectral power [15,17] and absolute slow spectral power [17] than men. After normalizing for total power, however, women had lower power over a small frequency range in the SWA (2–2.75 Hz) [15].

#### 2.5.3. Sigma Activity (11–16 Hz) and Sleep Spindles in NREM Sleep

Spectral power in the sigma range is associated with a specific sleep pattern: the sleep spindle. Sleep spindles correspond to bursts of sinusoidal waves at a frequency of 11–16 Hz, and are generated by an interaction between corticothalamic networks and the reticular nucleus of the thalamus [34]. Spectral power in sigma activity decreases with age, even after 60 years [17,28,35], and is associated with a gender difference, with older women having higher sigma spectral power than men [17]. Spindle density, amplitude and duration are reduced with age [36,37,38,39]. Age-related decline in density and amplitude is more prominent in anterior sites, while duration has a posterior prominence [38]. Spindle frequency is affected only minimally by aging [38]. It is especially the density of fast spindles (>13 Hz) that decreases with age, although their amplitude and duration also decrease [17]. 

Slow spindles (<13 Hz) are also affected by age, but to a lesser extent. Interestingly, slow spindle frequency slows down, while fast spindle frequency accelerates [17]. These age-related modifications in sleep spindle structure are the greatest during the final sleep cycles [40]. Spindle density declines after 60 years in men, while this decline was not observed in women [17,39]. On the other hand, spindle frequency in men increases after 60 years, and decreases in women [36,38]. Fast spindle density is lower in men, while there is no sex difference in slow spindle density or in spindle-SW coupling. Older individuals show greater decreases in slow spindle activity across the night, while younger individuals show greater increases in fast spindle activity across the night [39]. Higher spindle density in N2 is strongly associated with a shorter duration of N3 sleep and a greater duration of REM sleep, especially in older subjects [39]. A longitudinal study demonstrated reductions in spindle density (12–15%), amplitude (5–12%) and duration (2%) across the five years of follow-up [39]. 

Another marked age-related alteration is the magnitude of spindle–SW coupling for both fast and slow spindles, with spindles occurring earlier (on the rising phase in the SW cycle) in the SW in the elderly [17,35]. In young subjects, mean sleep spindle activity was nested just after the SW peak [35]. This coupled interaction between slow wave oscillations and sleep spindles during NREM is thought to assist in memory consolidation [41]. The modification of spindle structure and spindle-SW coupling could represent new potential markers of neurodegeneration [3,42,43].

## 3. Sleep Regulation: Interaction between Circadian and Homeostatic Drive

The sleep/wake cycle is controlled by two internal processes, the circadian and homeostatic drives [44], and the optimal sleep/wake timing, initiation and maintenance during 24 h, depending on their interaction.

### 3.1. Circadian Drive

In mammals, the circadian drive is generated by the circadian oscillator, which is located in the suprachiasmatic nucleus (SCN) of the hypothalamus [44]. The SCN displays circadian rhythms in electrical activity and a firing rate over 24 h, with high activity during the day and low activity during the night. The molecular principle of cellular circadian rhythmicity is driven by interlocked transcriptional/translational feedback loops composed of a subset of clock-related genes. In humans, the biological clock has an endogenous rhythmic property and oscillates over an intrinsic period that is slightly longer than 24 h (free-running period) [45]. Because the intrinsic period is not exactly 24 h, and to allow the daily rhythms of behaviors and physiological processes to be optimized and harmonized with the geo-physical environment, the biological clock is reset every day, and is entrained by what are known as zeitgebers [46] or synchronizers. The most important synchronizer in mammals is the light/dark cycle [47]. In humans, photic synchronizers are the natural day’s sunlight and night’s darkness, but also exposure to artificial lighting from electrical lighting and screens [48]. Light signals are detected by the retina [49], especially the melanopsin-containing intrinsically photosensitive retinal ganglion cells (ipRGCs) [50], and are transmitted to the biological clock via different pathways [51]. Non-photic synchronizers, especially physical activity, appear to have a chronobiotic effect, since they help realign the biological clocks in various animal [52], including humans [53].

During the biological day, the human circadian oscillator generates a circadian drive for wakefulness whose intensity is maximal at the end of the day, close to the onset of melatonin secretion. This maximal circadian drive for wakefulness is termed the “wake maintenance zone” or “forbidden zone of sleep”. Thereafter, the drive for wakefulness dissipates rapidly to make way for the circadian drive for sleep, which reaches its maximum at the end of the night close to the core body temperature minimum. When the circadian drive for sleep dissipates, the propensity to wake up appears [44]. Sleep timing and sleep and wake maintain depend mainly on circadian drive. 

### 3.2. Sleep Homeostatic Drive

The homeostatic oscillator triggers a homeostatic drive that corresponds to the physiological need for sleep, known as homeostatic sleep pressure, which increases during wakefulness and dissipates during sleep in an exponential manner [54]. The homeostatic process regulates the balance between the need for sleep and the time spent awake, so that the propensity to sleep gradually increases during wakefulness and decreases during sleep. The homeostatic process controls the amount and amplitude of SWA activity during slow wave sleep. Unlike the circadian drive, the cerebral connections and anatomical location of the sleep homeostat are still unknown. Homeostatic drive might be related to the accumulation (during wakefulness) or dissipation (during sleep) of a “sleep factor”, which enhances the activity of sleep-promoting neurons and reduces the activity of wake-promoting ones. 

The sleep factor that builds up in the brain during wakefulness and dissipates during sleep is not fully understood. Perhaps the best candidate is adenosine, whose level increases in the basal forebrain during time awake and decreases during sleep. These mechanisms might explain the action of adenosinergic drugs such as caffeine on sleep onset and SWA activity [55,56]. The A1 and A2A receptors are primarily involved in mediating the sleep-promoting effects of adenosine [57].

### 3.3. Sleep/Wake Regulation

There are several models for the regulation of the sleep-wake cycle. Current models all derive from the original Borbely model [58], which postulates that two processes, the homeostatic and the circadian processes, are independent, and that their effects are additive. In summary, during a normal day, the buildup of sleep pressure is counteracted by the circadian drive for wakefulness, especially during the wake maintenance zone, in order to extend the period of wakefulness. After this specific zone, the combination of higher homeostatic drive and rapid dissipation of circadian drive for wakefulness leads to the propensity to fall asleep. After sleep onset, the rapid dissipation of homeostatic drive is counteracted by the circadian drive for sleep in order to extend the nocturnal sleep period. The dissipation of the circadian drive for sleep combined with the dissipation of the circadian and sleep-dependent promotion of rapid eye movement (REM) sleep leads to the propensity to wake up. Homoeostatic and circadian drive interact to facilitate consolidated wakefulness throughout the day and consolidated sleep throughout the night. This consolidation of wakefulness and sleep is effective only if the circadian drive is strong enough. This two-process model has been complemented and extended by neurophysiologic data. For example, the Phillips-Robinson model [59,60] is based on the mutually inhibitory sleep-promoting VLPO (GABAergic) and the wake-promoting monoaminergic hypothalamic and brainstem neuronal populations (MA). The model produces flip-flop dynamics between sleep and wake that are driven by homeostatic/circadian processes and orexinergic neurons. During the day, the circadian drive produced by the SCN inhibits VLPO and activates the orexinergic neurons of the lateral hypothalamic area (Orx). In the evening, circadian drive decreases and the strength of homeostatic drive is maximal, disinhibiting VLPO, which inhibits Orx and therefore inhibits MA, finally triggering sleep onset and stabilizing sleep episodes. At the end of the night, the strength of homeostatic drive is the lowest, VLPO is not activated, circadian drive begins to inhibit VLPO and to activate Orx, and waking is triggered. In this model, changing homeostatic parameters affect sleep duration, with reduced homeostatic drive tending to result in later bedtimes rather than earlier wake times, while changing circadian parameters change sleep timing.

There hypothalamic-pituitary-adrenal (HPA) axis and sleep regulation might be related (for review see [61]). On the one hand, slow wave sleep has an inhibitory influence on the HPA axis, whereas stage 1 NREM (N1) or arousal has an excitatory influence on it. Sleep duration influences mean 24-h plasma cortisol levels: short sleep duration is associated with high mean 24-h plasma cortisol levels and vice-versa. Moreover, sleep fragmentation is associated with significant increases in plasma cortisol levels. On the other hand, activation of the HPA axis by stress states or administration of glucocorticoids induce suppression of sleep, especially REM sleep, and can lead to arousal and insomnia. In older adults, a higher sensitivity to the resulting arousal-producing stress hormones may initiate or perpetuate insomnia symptoms, i.e. difficulty in sleep onset and maintaining sleep. This observation is consistent with the hypothesis of central nervous hyperarousal in insomnia. The hyperarousal model posits that insomnia is due to an ustable state, such as weak activation of the VLPO, which activates sleep via GABAergic cells, and hyperactivation of the ascending reticular activating system, which leads to arousal via orexinergic cells. In addition to the relationship between the HPA axis and sleep homeostasis, glucocorticoids might also modulate sleep indirectly by influencing the activity of the circadian clock. System glucocorticoids influence peripheral clocks at multiple sites and are key in the resetting of the circadian system after a phase shift.4. Age-Related Changes in Homeostatic and Circadian Drive.

### 3.4. Circadian Pacemaker and Rhythms

Even though the size, volume and number of SCN cells do not seem to change with age, the reduction of amplitude in firing activity [62] suggests alterations in the properties of SNC, neuronal circuitry and clock genes with age [63]. The decrease in the activity of SCN cell firing is accompanied by a reduction in arginine vasopressin and vasoactive intestinal peptide expression, fewer GABAergic synapses and modifications in the electrical properties of membranes and synaptic connectivity (for review: [64]). The core clock machinery shows alterations with age in the SCN of middle- and old-aged rodents (for review: [63]). Impaired melatoninergic retro-control of the SCN is thought to be related to decreased melatonin receptor 1 expression with age. 

The major age-related modifications of circadian rhythms are the phase advance (earlier phase of circadian rhythms and earlier chronotype), and dampening of the amplitude of rhythm is reported in some studies [65] but not all [66]. The phase advance is not related with the shortening of the endogenous circadian period [67]. The endogenous circadian period of sleep is stable, and does not change with age [45]. These age-related modifications could be due to the flattened and phase-advanced expression of specific clock genes [68] in the prefrontal cortex. A gender-dependent dampening of amplitude may occur, with a reduction in circadian amplitude, predominantly in elderly men [69]. The age-related advance of the sleep-wake cycle could also be related to changes affecting the synchronization the circadian clock [70] or the homeostatic drive for sleep [29].

### 3.5. Sleep/Wake Regulation

Forced desynchronization experiments [29] (people living on imposed sleep/wake of 28 h during for at least one month) have shown that the circadian process keeps robustly to the 24 h period despite desynchronization. However, there is a decrease in the propensity to fall asleep in the early morning. On the other hand, the homeostatic process is always operational but reduced. The amount of slow-wave sleep is reduced, and the duration of sleep is reduced regardless of the circadian time. Sleep consolidation is also degraded with a strong increase in arousals in the second part of sleep, especially when sleep occurs after the melatonin peak. These results demonstrate that sleep consolidation in elderly subjects is sensitive to the poor adjustment-synchronization of the sleep-wake cycle with circadian rhythms (body temperature and melatonin). Moreover, they demonstrate that elderly subjects are not able to maintain their sleep at the end of the night after the melatonin peak or temperature nadir, unlike younger subjects. Age-related sleep changes are therefore likely related to the interaction between the reduction in sleep pressure, i.e., the homeostatic process, and the reduction in the strength of the circadian signal, especially late at night or early in the morning [29]. 

The age-related phase advance in sleep-wake timing cannot be attributed to a shortening of the circadian period or to an advance of circadian phase, but rather depends on an inability to sustain sleep in early morning [71]. Using an experimental protocol that allows the effect of low or high homeostatic pressure to be measured at all positions of the circadian phase, Cajochen’s team demonstrated that it is primarily the interaction of the circadian and homeostatic processes, which plays a role in age-related sleep disorders, more than the reduction in the homeostatic process alone, which would be intact in the elderly [72]. On the other hand, in contrast to forced desynchronization experiments, this type of protocol has demonstrated a decrease in the pressure of arousal induced by the circadian process at the end of the day. The circadian signal is attenuated in the elderly, and thus weaker in opposing the homeostatic sleep pressure build up during wakefulness, so the elderly may experience higher sleep pressure during wakefulness, i.e., faster wake-dependent homeostatic increase in sleep pressure during daytime. 

A study investigating recovery sleep after the selective deprivation of slow-wave sleep demonstrated that age-related sleep changes in particular reduced slow-wave sleep, and a smaller rebound in SWA after selective deprivation reduced the propensity to fall asleep during the day, reflecting more of a reduction in sleep need than a weakness in the homeostatic process [26]. Older subjects should therefore feel less sleepy during the day than younger subjects, which would explain why older subjects are less affected by the duration of increased wakefulness, i.e., by sleep deprivation. 

The reduction in the homeostatic process with age could be linked to a strong reduction in A1 adenosine receptors, despite higher levels of adenosine in elderly subjects [73]. Therefore, the reduction in A1 receptors may decrease sensitivity to higher extracellular adenosine concentrations, despite the increased build-up of adenosine levels reflecting a higher sleep need in older adults [3]. In contrast with Dijk’s hypothesis that sleep need decreases with age, Mander et al. [3] suggested that the sleep need remains in older subjects, but that capacity to regulate and/or generate sleep declines. 

The extended Phillips-Robinson model demonstrates that age-related sleep changes depend on a reduction in circadian amplitude, resulting in earlier sleep timing and reduced average homeostatic sleep pressure, as well as a smaller difference between wake time and the endogenous circadian phase, i.e., phase angle difference. Using the Phillips-Robinson model, Skeldon [74] attempted to explain age-related changes in sleep. From adolescence to adulthood, the reduction in sleep duration and late bedtime are explained by a progressive reduction in circadian amplitude and sleep pressure. After 20 years of age, the reduction in circadian amplitude might be the most important modification, and would explain the advance in times at which people rise and go to bed. However, the reduction in the homeostatic process alone would also lead to a shorter sleep duration, and thus to earlier awakening due to an advanced exposure to light, which would modify the amplitude of the circadian rhythms. Changes in orexin levels have not yet been tested, but the hypothesis of a loss of wake-promoting orexin neurons in older subjects has been put forward (see Carrier [5] for review). 

The entrainment phase angle characterizes the adjustment/synchronization of the sleep-wake cycle with circadian rhythms and with the outside world. The phase angle represents the time interval between the phase of a circadian marker (thermal minimum or onset of melatonin secretion) and the beginning, middle or end of sleep. This temporal relationship is important because it is a true reflection of the length of the endogenous period. The phase angle is different between young and old subjects [75]. Elderly subjects wake up just after the thermal minimum, and when their melatonin level is still relatively high, in contrast to younger subjects. Older subjects wake up earlier in the circadian profile than younger subjects. It also appears that age is associated not only with a phase advance in wake/sleep schedules and circadian rhythms, but also with a change in the phase relationship between the sleep-wake cycle and circadian rhythms.

Briefly, the physiological causes of age-related sleep changes are not yet known because of the complexity of the regulation of the sleep/wake system. Moreover, the decline in SWA is associated with atrophy in the prefrontal cortex, as indicated by reduced gray matter volume and cortical thickness (See Mander [3], Dubé [33] for review).

The visceral theory of sleep could provide a new perspective on many aspects of sleep in aging. The theory is based on the informational approach to understanding the function of sleep [76,77]. It postulates that the central nervous system during sleep is involved in the process of visceral regulation. The system, including all cortical areas, is thought to switch from the processing of exteroceptive information arising from various modalities to the processing of interoceptive information obtained from dif-ferent visceral systems. This change in cortical afferentation during sleep involves the simultaneous change in the direction of the flow of efferent cortical information. In wakefulness, the flow is towards the structures involved in the organization of behav-ior. During sleep, it is redirected to the structures subsuming visceral regulation. Analysis of the visceral hypothesis of sleep shows that many disorders involving the sleep-wake cycle can be explained by asynchronous switches of cortical afferent and efferent information flow. 4.3. Sensitivity to Light and Circadian Synchronization.

The most frequently described rhythmic change observed with aging is a phase advance of the circadian timing system and of the sleep-wake cycle [65,78]. It has long been—and still is—attributed to a shortening of the circadian period, but this hypothesis has been refuted in highly controlled studies conducted in healthy individuals, showing that the period is the same in young and older individuals [45]. Therefore, the age-related advance of the sleep-wake cycle relate to other mechanisms, either involved in the circadian or the homoeostatic processes, or both.

Physiological correlates of the circadian changes have been described at the input level in the eye [79,80,81], at the internal clock level in the SCN [82,83,84,85], and at the output level in different brain structures, including the melatonin-releasing pineal gland [86,87].

At the input level, a number of responses to light have been shown to decrease with age, including melatonin suppression, the pupillary light reflex and circadian phase-shifting [70,80,88,89,90,91,92]. The reduced sensitivity to light is thought to involve optical and/or neural changes [93,94]. The age-related reduced pupil size [89,95] and increased ocular lens absorption [96,97] both lead to a decreased retinal illumination, in particular in the blue range of the visible light spectrum to which non-visual responses is particularly sensitive to, due to the melanopsin ganglion cells [48,98,99,100,101,102]. However, studies in older individuals have reported conflicting results, suggesting that age-related physiological changes do not necessarily lead to reduced non-visual effects of light. Indeed, a lower circadian sensitivity to fluorescent light has been found in older individuals at moderate intensities (100–1000 lux) [70], but not at higher (>2000 lux) nor at lower light intensities, even over different circadian phases [70,88,103,104]. 

A number of studies have been conducted using monochromatic light exposures. A study reported that short wavelengths (456 nm) induced smaller melatonin suppression in the aged but not long wavelengths (548 nm) [105]. Another study from the same group found that short wavelength lights induced similar circadian phase advances in young and older subjects [106]. A more recent study investigating melatonin suppression in response to a 60-min light pulse found that spectral sensitivity was shifted toward longer wavelengths in older adults, but that amplitude of the suppression was similar in young and older adults [80]. The discrepancies between those findings are unclear, but are likely related to the intensities used in the studies given the non-linear relationships linking both intensity of light exposure to non-visual responses [101,107,108,109,110]. Indeed, if high light intensities are used and saturate the effect (higher intensities not inducing higher effects), then similar responses would be expected to be found in aged and young subjects even if retinal exposure was reduced in the older individuals compared to the younger. A similar mechanism may also apply to responses as a function of duration of light exposure [110,111], as long saturating durations would be expected to induce similar responses in young and older individuals, and differences more likely to be found at short durations due to spectral tuning of sensitivity to light [112]. Other explanations of the conflicting results found in the literature may relate to the large differences in light sensitivity across non-visual responses recently described [110], or to the high interindividual variability within single responses [102]. The mechanisms involved remain to be clarified, but the result that the increased lens filtering occurring with aging does not lead to a proportional decrease in non-visual sensitivity to light suggests that non-visual responses to light undergo adaptive or compensatory mechanisms during healthy aging [80].

## 4. Daytime Sleepiness and Vulnerability to Sleep Deprivation

### 4.1. Daytime Sleepiness

It is commonly accepted that older subjects take more daytime naps than younger subjects. Yoon [113] demonstrated that napping episodes were slightly more frequent in healthy older adults, but the daily duration of napping is similar in young and old subjects. Older adults nap more in the evening, whereas young adults nap more in the afternoon. In healthy subjects, napping is not associated with increased levels of objective daytime sleepiness. Unexpectedly, sleep latencies measured during a multi sleep latency test in older and middle-aged subjects were significantly longer than those in young subjects, confirming an age-related reduction in daytime sleep propensity [26]. The mean sleep latency in older subjects tended to be longer than in middle-aged subjects, especially the sleep latencies of the morning tests. On the other hand, a study on naps revealed that the circadian wake-promoting signal in the evening hours was weaker in older participants, with higher subjective sleepiness ratings and more sleep occurring during the wake maintenance zone in the late afternoon [114] in older than in young adults [115]. Thus, reduced circadian wake promotion during the biological day might promote daytime naps in older adults [116]. Excessive daytime sleepiness in the elderly, i.e., the inability to maintain wakefulness and alertness during the major waking episodes of the day, usually co-exists with the use of substances or medications known to induce sleepiness and with several adverse health conditions, such as sleep disorders, comorbid medical diseases, psychiatric disorders and physical pain. While excessive daytime sleepiness is not a part of normal aging, it may be a signal or symptom of certain diseases [117,118,119].

### 4.2. Vulnerability to Sleep Deprivation

Extended wakefulness is known to impair neurobehavioral performance such as attention [120,121]. In some studies, however, an age-related inter-individual vulnerability has been described. Studies have demonstrated that acute sleep loss has a less detrimental effect on performance on cognitive tasks in elderly people than in younger ones [117,122,123,124,125,126,127,128,129,130,131]. Young people show a higher performance decrement than older people during the biological night (24 h–8 h) [117,126,130,132]. Thus, sleep pressure-related slowing in the young ‘‘makes them old’’, while older people, notwithstanding cognitive slowing [133], are less susceptible to circadian and wake-dependent performance decrements. This lower age-related vulnerability to extended wakefulness seems predominantly due to an attenuated circadian regulation on performance in older subjects [72,118,127,130,134], especially in the late biological night [130]. Regardless of age, there is inter-individual vulnerability to the deleterious effects of sleep deprivation on neurobehavioral performance due to the chronotype or *PER3* gene polymorphism [135,136].

In a forced desynchrony study, Silva [118] demonstrated that reaction time on a sustained attention task and the ability to perform mental arithmetic were less deteriorated by the cumulative effects of repeated exposure to adverse circadian phase in older subjects than in young ones. This suggested that there are age-related changes in the circadian promotion of alertness, in the wake-dependent decline in alertness, and/or in the interaction between the circadian and homeostatic processes.

By contrast, studies have shown a greater or similar effect of sleep deprivation on vigilance in young and old people [137,138,139]. This lack of consensus is probably due to the characteristics of the tasks used, the types of variables (speed- or error-related component) and circadian/homeostatic manipulations in various protocols. Aging could be a protective factor against the effects of extended wakefulness, especially regarding sustained attention failures due to attenuation of sleep pressure with duration of time awake [130].

A few studies have examined the contribution of the circadian system and sleep pressure influences on executive performance as a function of age. Executive functions (i.e., higher-order cognitive processes) are essential for behavioral adjustment to ongoing goals when facing novel or complex situations. Executive functions influence other cognitive processes, and their integrity is central in aging to remain adapted to the environment and live autonomously. Inhibition of action is a major component of executive control to adapt behavior (i.e., the suppression of prepotent or automatic actions that have become inappropriate) when unexpected changes in the environment occur [140]. There is a lack of consensus on the effects of sleep deprivation on executive functions. Some studies reported the deleterious effect of sleep deprivation on decision-making [141] and more largely on executive performance [130,142,143,144,145,146]. Conversely, others found no impact of sleep deprivation on executive functioning [147,148,149]. Here again, the effects of time of day and sleep loss depend on the specific component of executive functioning tested, and on the paradigm used [150].

Using an experimental protocol that allows the effect of low or high homeostatic pressure to be measured at all positions of the circadian phase, Sagaspe et al. [130] determined the age-related evolution of executive function, inhibitory motor control and sustained attention. Under high homeostatic pressure, i.e., total sleep deprivation, simple reaction time performance decreased in both young and older groups especially during the biological night, but even more in young participants as expected. Moreover, young and older individuals experienced difficulty in inhibiting an inappropriate prepotent response (i.e., inhibition failure) and difficulty in responding accurately to appropriate stimuli (i.e., sustained attention failure) during and after a night of sleep deprivation, although the effect was greater in the young participants. Under low homeostatic pressure with multiple naps during the experiment, the circadian influence on reaction time performance in the elderly was found to be attenuated. These results confirm that the age-related lower vulnerability to extended wakefulness seems predominantly due to an attenuated circadian regulation on reaction time performance in the elderly, especially during the biological night. Older people demonstrated not only an attenuation of the circadian influence on speed-related processing, but also a reduction in sleep pressure with duration of time awake on sustained attention error-related processing. Overall, the study showed that error-related processing in a behavioral inhibition task does not seem to be regulated by the circadian component, contrary to speed-related processing, and that it depends principally on the effect of increasing sleep pressure with duration of time awake [130,146]. Homeostatic sleep pressure seems to be lower in older people, making them less vulnerable to attentional failure after a night of sleep deprivation [130,151].

Age-related cognitive decline arises from alterations in brain structure, as well as in sleep-wake regulation. fMRI investigations have shown that homeostatic sleep pressure exerts an influence on sustained attention-related cerebral activity in the hypothalamus, which is itself involved in the regulation of the circadian wake promoting signal [152,153], as is the endocannabinoid system [154]. Frontal brain regions are particularly prone to both the aging process and misalignment between the circadian and homeostasis processes, even though evidence indicates dissociation between these influences on frontal-activation -related executive functions [155,156,157]. The impact of age-related changes in the circadian and homeostatic processes on the cerebral activity subtending waking performance remains underexplored [116]. Recent studies have shown that preserved wake-dependent cortical excitability dynamics predict cognitive fitness in aging, particularly in the executive domain, independently from modifications in brain structural integrity that can ultimately lead to dementia [158,159].

The bottom-up theory thinking this that temporal changes in neurobehavioral performance during sleep deprivation and across time-on-task is essentially local and use-dependent in nature. It hypothesizes that neuronal groups involved in performing a given task will fall asleep locally as a homeostatic consequence of sustained use, thereby interrupting information processing and leading to performance impairment [160,161]. Sleep is also regulated at a more local level in the brain: NREM sleep and wakefulness can occur simultaneously in different parts of the cortex in humans [162]. Recently, McKillop et al. [163] report that older mice does not lead to marked changes in vigilance state-related local neural activity, despite pronounced global changes in the daily amount and distribution of waking and sleep. These results suggest that older mice have an intact capacity to generate slow waves and a homeostatic response to sleep loss at the local cortical level, while the global sleep dynamics appear to be profoundly disrupted. 

Further studies designed to precisely quantify circadian and homeostatic influences under highly controlled conditions (i.e., constant routine protocol, forced desynchrony protocol) on performance are necessary. To better understand the influence of sleep/wake regulation that contributes to simple and complex human cognitive processes is a key challenge for the cognitive neurosciences. Furthermore, the predictive value of sleep-wake regulation for cognitive decline and the risk of developing dementia remains to be investigated in longitudinal protocols.

The age-related alterations in circadian and homeostatic sleep regulation negatively impact daytime cognitive performance. Furthermore, the impact of the circadian/homeostatic interaction on modulating sleep and cognition is lower in older healthy people [116]. The cerebral systems involved in waking may modulate cognition through their connection with the cerebral cortex. Optimistically, cognitive reserve could serve as a compensatory factor for age-related changes in sleep and cognitive decline [156].

## 5. Conclusions

The most important sleep disturbances that continue to worsen after 60 years of age involve sleep timing, especially early wake time (phase advance), sleep maintenance and a reduced amount of sigma activity during NREM sleep associated with modifications of sleep spindle characteristics and spindle–SW coupling. After this age, there is a gender-dependent deterioration in sleep duration (men sleeping less than women), percentage of sleep stage N3 (men having less) and SWA (men having less). On the other hand, neither daytime wake level nor daytime sleepiness are modified with age. Older adults have less cognitive impairment during extended wakefulness than younger ones. These age-related sleep modifications could be due to a reduced homeostatic drive, and consequently to a reduced need for sleep, an attenuated circadian drive and/or modifications in the interaction between the circadian and homeostatic processes. The extent of these modifications and interactions as people age is still uncertain. Nevertheless, the alteration of the subcortical structures involved in generating circadian and homeostatic drive and of the connections to the cerebral cortex with age is a consequence of age-related sleep disruption [116]. The mechanisms underlying these age-related differences in sleep are of great interest to the scientific community [5]. In the context of geriatric research, a growing body of evidence is linking normal age-related changes in sleep with many adverse health outcomes, especially a decline in cognition in older adults [43,119]. Age-related changes in the sleep-wake cycle are associated with current cognitive status [164,165], but also predict future cognitive trajectories such as dementia [166,167,168,169]. Understanding the respective contribution of the circadian and homeostatic processes in the regulation of neurobehavioral function with aging is a challenge for improving health, managing cognitive decline and developing early sleep-wake interventions [158,170].

## Data Availability

Not applicable.
